# Association between somatostatin analogues and diabetes mellitus in gastroenteropancreatic neuroendocrine tumor patients: A Surveillance, Epidemiology, and End Results‐Medicare analysis of 5235 patients

**DOI:** 10.1002/cnr2.1387

**Published:** 2021-04-09

**Authors:** Katherine Ni, Jeong Yun Yang, Kiwoon Baeg, Amanda C. Leiter, Grace Mhango, Emily J. Gallagher, Juan P. Wisnivesky, Michelle K. Kim

**Affiliations:** ^1^ Department of Medicine Icahn School of Medicine at Mount Sinai New York New York USA; ^2^ Division of Gastroenterology Icahn School of Medicine at Mount Sinai New York New York USA; ^3^ Division of Endocrinology Icahn School of Medicine at Mount Sinai New York New York USA; ^4^ Tisch Cancer Institute at Mount Sinai Icahn School of Medicine at Mount Sinai New York New York USA; ^5^ Division of Pulmonary, Critical Care, and Sleep Medicine Icahn School of Medicine at Mount Sinai New York New York USA

**Keywords:** cancer survivorship, digestive cancer, epidemiology, neuroendocrine tumor, SEER, somatostatin analogue

## Abstract

**Background:**

Gastroenteropancreatic neuroendocrine tumors (GEP‐NETs) are increasingly common malignancies and tend to have favorable long‐term prognoses. Somatostatin analogues (SSA) are a first‐line treatment for many NETs. Short‐term experiments suggest an association between SSAs and hyperglycemia. However, it is unknown whether there is a relationship between SSAs and clinically significant hyperglycemia causing development of diabetes mellitus (DM), a chronic condition with significant morbidity and mortality.

**Aim:**

In this study, we aimed to compare risk of developing DM in patients treated with SSA vs no SSA treatment.

**Methods and Results:**

Using the Surveillance, Epidemiology, and End Results (SEER) database and linked Medicare claims (1991‐2016), we identified patients age 65+ with no prior DM diagnosis and a GEP‐NET in the stomach, small intestine, appendix, colon, rectum, or pancreas. We used *χ* ^2^ tests to compare SSA‐treated and SSA‐untreated patients and multivariable Cox regression to assess risk factors for developing DM. Among 8464 GEP‐NET patients, 5235 patients had no prior DM and were included for analysis. Of these, 784 (15%) patients received SSAs. In multivariable analysis, the hazard ratio of developing DM with SSA treatment was 1.19, which was not statistically significant (95% CI 0.95‐1.49). Significant risk factors for DM included black race, Hispanic ethnicity, prior pancreatic surgery, prior chemotherapy, tumor size >2 cm, pancreas tumors, and higher Charlson scores.

**Conclusion:**

DM was very common in GEP‐NET patients, affecting 53% of our cohort. Despite prior studies suggesting an association between SSAs and hyperglycemia, our analysis found similar risk of DM in SSA‐treated and SSA‐untreated GEP‐NET patients. Further studies are needed to better understand this relationship. As NET patients have increasingly prolonged survival, it is crucial to identify chronic conditions such as DM that these patients may be at elevated risk for.

## 1 INTRODUCTION

Gastroenteropancreatic neuroendocrine tumors (GEP‐NETs) are an increasingly common group of malignancies, with diagnosis rates increasing by more than sixfold in the last four decades and continuing to rise.^1^, ^2^ Even in patients who present with advanced stage tumors, the long‐term prognosis is relatively favorable, with median survivals of 5 years or more.^3^, ^4^ A common first‐line treatment for NETs is a somatostatin analogue (SSA), such as octreotide or lanreotide. Given the often‐prolonged duration of treatment with SSAs and long survival of patients with GEP‐NETs, it is important to understand the potential long‐term effects of these treatments.

Diabetes mellitus (DM) is a serious condition that has been reported in approximately 21.4% of all individuals over age 65.^5^ DM is associated with significant complications and morbidity, decreased quality of life, and increased mortality.^6^, ^7^, ^8^, ^9^, ^10^

SSAs have been shown to alter glucose homeostasis. In clinical trials of the octreotide acetate long‐acting injection in NET patients, hyperglycemia was observed in 27%.^11^ Additionally, in small studies in non‐NET patients, intravenous or subcutaneous octreotide was found to be effective in treating sulfonylurea‐induced hypoglycemia in the acute setting,^12^, ^13^ thought to be via octreotide's inhibition of insulin release from pancreatic beta cells. However, the effects of SSAs on glucose regulation are complex. While SSAs inhibit insulin and glucagon‐like peptide 1 (GLP‐1) secretion, which increases blood glucose; they also inhibit secretion of the counterregulatory hormones, growth hormone and glucagon, thereby reducing insulin resistance and blood glucose levels.^14^, ^15^, ^16^ Though less common than instances of hyperglycemia, hypoglycemia was observed in 4% of NET patients in the octreotide clinical trials.^11^

In NET patients, small studies have suggested an association between GEP‐NETs and metabolic syndrome^17^ and higher fasting glucose,^18^ but these initial studies showed no statistically significant difference in these measures in patients treated with SSAs. Overall, although hyperglycemia is more often suggested than hypoglycemia, the net effect of SSAs on glucose homeostasis in GEP‐NET patients is not well‐understood. Additionally, whether SSAs predict clinically significant and long‐term hyperglycemia causing subsequent diabetes mellitus has not been studied.

Therefore, our primary aim in this retrospective study is to determine whether SSA treatment independently increases risk of developing DM in GEP‐NET patients.

## 2 METHODS

### 2.1 Study population

The study was approved by the Icahn School of Medicine Institutional Review Board: Study IRB‐20‐03072.

Using the Surveillance, Epidemiology, and End Results (SEER) database and linked Medicare claims from 1991 to 2016, we identified individuals over age 65 diagnosed with a single GEP‐NET in the stomach, small intestine, appendix, colon, rectum, or pancreas, and no prior DM diagnosis. We excluded patients with a diagnosis of diabetes predating their GEP‐NET diagnosis date and patients for whom a GEP‐NET was diagnosed on autopsy. We excluded patients without Medicare Part B Coverage, which covers outpatient care and certain outpatient medications, and patients enrolled in a health maintenance organization (HMO), as Medicare does not have full claims records for these individuals. Participants were followed until date of death or until the end of claims reporting on December 31, 2016.

Variables of interest obtained from the SEER‐Medicare database included age, race/ethnicity, tumor size, tumor grade, and tumor stage. Participants were defined as having the clinical symptoms of carcinoid syndrome if they had two or more diagnosis codes for “diarrhea,” “flushing,” or “carcinoid syndrome” using International Classification of Diseases, Ninth Revision (ICD‐9) diagnosis codes. Prior NET treatments with SSAs (octreotide or lanreotide), pancreatic surgery, radiation, or chemotherapy were identified by ICD‐9 diagnosis codes and Healthcare Common Procedure Coding System (HCPCS) codes, billing codes which identify services, procedures, and supplies. All treatments were considered binary variables, such that patients who received a treatment for any period of time were treated equally as having received the treatment. Charlson comorbidity index was calculated using ICD‐9 code claims preceding the date of NET diagnosis for each patient. Patients were defined as having developed DM by determining the date of the first occurrence of an ICD‐9 code for DM across all of an individual's claims after the date of GEP‐NET diagnosis.

The primary outcome was the risk of developing DM in SSA‐treated vs SSA‐untreated GEP‐NET patients. Secondary outcomes were identification of other significant predictors of developing DM in adjusted analysis.

### 2.2 Statistical analysis

We compared patients who received SSA with those who did not receive SSA using chi‐squared tests. We used a multivariable Cox proportional hazards model to identify independent risk factors for developing DM. In this time‐to‐event analysis, the target event was the time of DM diagnosis, and the time origin was the GEP‐NET diagnosis date. Receipt of SSA treatment was analyzed as a time‐dependent variable, such that the value of the binary “received SSA” variable is reassigned at each time point that there is an event (ie, a diagnosis of diabetes). This accounts for the possibility that SSA may have been started at varying time points before or after DM diagnosis. Individuals who had no DM diagnosis by the date of last follow‐up were treated as censored observations. Kaplan–Meier survival analysis was performed to compare survival in DM patients vs non‐DM patients, for the entire cohort of NET patients, and for subgroups of pancreatic primary tumors compared to tumors of other primary sites. A *P*‐value of <.05 was considered statistically significant. All statistical analyses were performed using SAS (Cary, North Carolina).

## 3 RESULTS

We identified 8464 patients with a pathologically confirmed GEP‐NET from the SEER database and linked Medicare claims from 1991 to 2016. Of these, 3229 (38% of all GEP‐NET patients) had a diagnosis of DM predating their NET diagnosis and were excluded from the main analysis. The remaining 5235 patients were included for analysis. Of these patients, the vast majority never received SSA, with only 784 (15%) receiving SSA treatment after NET diagnosis.

The median age of this cohort was 72, with even distributions of men and women (49.4% men). The large majority of patients (77.1%) were non‐Hispanic white, 10.5% were non‐Hispanic black, 5.4% were Hispanic, and 5.5% were Asian and Pacific Islander (API). Small intestine tumors were most common, making up 35.6% of all tumors included, followed by rectal (18.3%), pancreatic (17.4%), colonic (14.8%), gastric (9.7%), and appendiceal (4.3%) tumors. Tumor stage was bimodal, with a high frequency of stage 1 (40.4%) and stage 4 (33.1%) tumors, and just 9.8% stage 2 and 16.7% stage 3 tumors. Over half (55.3%) of tumors were grade 1 (well‐differentiated), while 16.8% were grade 2 (moderately differentiated), and 27.9% were grade 3 (undifferentiated or poorly differentiated).

In an unadjusted comparison, 30.6% of patients who received SSA developed DM after NET diagnosis, vs 24.2% of patients who did not receive SSA (*P* = .0002) (Table 1). Patients who received SSA were significantly more likely to be younger (age 65‐69 group), have received chemotherapy or prior radiation therapy, have clinical symptoms of carcinoid syndrome, and have a larger and later stage tumor. There were also statistically significant differences in the distributions of tumor site, race/ethnicity, and Charlson comorbidity score between groups that did and did not receive SSA.

**TABLE 1:** Baseline characteristics of GEP‐NET patients without prior diabetes mellitus

Variable		No SSA treatment N = 4451	Received SSA treatment N = 784	*P*‐value
		N (column %)		
Age	65–69	1739 (39.1%)	340 (43.4%)	**.0002**
	70‐74	1026 (23.1%)	201 (25.6%)	
	75‐79	765 (17.2%)	133 (17.0%)	
	80+	921 (20.7%)	110 (14.0%)	
Gender	Male	2210 (49.7%)	378 (48.2%)	.46
Race/ethnicity	Non‐Hispanic white	3365 (75.6%)	673 (85.8%)	**<.0001**
	Non‐Hispanic black	493 (11.1%)	59 (7.5%)	
	Hispanic	248 (5.6%)	32 (4.1%)	
	Asian/Pacific Islander	268 (6.0%)	18 (2.3%)	
Tumor size	<1 cm	796 (17.9%)	20 (2.6%)	**<.0001**
	1‐2 cm	837 (18.8%)	159 (20.3%)	
	>2 cm	1334 (30.0%)	373 (47.6%)	
	Unknown	1484 (33.3%)	232 (29.6%)	
Stage	I	1905 (46.4%)	53 (7.1%)	**<.0001**
	II	416 (10.1%)	60 (8.0%)	
	III	676 (16.5%)	136 (18.2%)	
	IV	1108 (27%)	498 (66.7%)	
Histological grade	Well differentiated	1099 (24.7%)	237 (62.5%)	**<.0001**
	Moderately differentiated	311 (7.0%)	95 (25.1%)	
	Poorly differentiated and undifferentiated	626 (14.1%)	47 (12.4%)	
	Unknown	2415 (54.3%)	405 (51.7%)	
Primary site	Appendix	212 (4.8%)	12 (1.5%)	**<.0001**
	Colon	704 (15.8%)	71 (15.4%)	
	Pancreas	719 (16.2%)	193 (24.6%)	
	Rectum	929 (20.1%)	27 (3.4%)	
	Stomach	470 (10.6%)	35 (4.5%)	
	Small intestine	1417 (31.2%)	446 (56.9%)	
Prior pancreatic surgery		257 (5.8%)	46 (5.9%)	.92
Carcinoid syndrome		618 (13.9%)	359 (45.8%)	**<.0001**
Prior chemotherapy		430 (9.7%)	395 (50.4%)	**<.0001**
Prior radiotherapy		233 (5.2%)	85 (10.8%)	**<.0001**
Modified Charlson comorbidity index	0	2457 (55.2%)	553 (70.5%)	**<.0001**
	1–2	1021 (22.9%)	138 (17.6%)	
	>3	210 (4.7%)	13 (1.67%)	
	Unknown	763 (17.1%)	80 (10.2%)	
Developed diabetes mellitus		1079 (24.2%)	240 (30.6%)	**.0002**

Compared to those who did not develop DM, individuals who developed DM were more likely to have received SSA, chemotherapy, or pancreatic surgery. There were also statistically significant differences in age, race/ethnicity, stage, grade, and primary site distributions (Table 2). Additionally, in a univariate Cox regression model, treatment with SSA with time‐dependent adjustments showed a hazard ratio (HR) of 1.31 for developing DM, which was statistically significant (95% CI 1.09‐1.58, *P* = .004).

However, in a multivariable model with analysis of SSA with adjustment for other covariates, SSA‐treated patients had similar risk of DM as SSA‐untreated patients (HR 1.19; 95% CI 0.95‐1.49) (Table 3). Patient factors associated with increased DM risk included Hispanic ethnicity (HR 1.58; 95% CI 1.25‐2.01) and black race (HR 1.58; 95% CI 1.33‐1.89). Age was not independently associated with development of DM. A sub‐analysis excluding pancreatic tumors showed consistent results, with no significant difference in DM risk in SSA‐treated and SSA‐untreated individuals (HR 1.14; 95% CI 0.87‐1.49).

**TABLE 2:** Characteristics of GEP‐NET patients, by development of diabetes mellitus during study period

Variable		No diabetes mellitus N= 3916	Developed diabetes mellitus N= 1319	*P*‐value
		N (column %)		
Age	65‐69	1496 (38.2%)	583 (44.2%)	**<.0001**
	70‐74	913 (23.3%)	314 (23.8%)	
	75‐79	675 (17.2%)	223 (16.9%)	
	80+	832 (21.3%)	199 (15.1%)	
Gender	Male	1965 (50.2%)	623 (47.2%)	0.06
Race/ethnicity	Non‐Hispanic white	3092 (79.0%)	946 (71.7%)	**<.0001**
	Non‐Hispanic black	373 (9.5%)	179 (13.6%)	
	Hispanic	192 (4.9%)	88 (6.67%)	
	Asian/Pacific Islander	204 (5.2%)	82 (6.22%)	
Tumor size	<1 cm	612 (15.6%)	204 (15.5%)	.57
	1‐2cm	745 (19.0%)	251 (19.0%)	
	>2cm	1294 (33.0%)	413 (31.3%)	
	Unknown	1265 (32.3%)	451 (34.2%)	
Stage	I	1369 (37.4%)	589 (49.4%)	**<.0001**
	II	343 (9.4%)	133 (11.2%)	
	III	623 (17.0%)	189 (15.9%)	
	IV	1325 (36.2%)	281 (23.6%)	
Histological grade	Well differentiated	1022 (26.1%)	314 (23.8%)	**<.0001**
	Moderately differentiated	323 (8.3%)	83 (6.3%)	
	Poorly differentiated and undifferentiated	584 (14.9%)	89 (6.8%)	
	Unknown	1987 (50.7%)	833 (63.2%)	
Primary site	Appendix	185 (4.7%)	39 (3.0%)	**<.0001**
	Colon	632 (16.1%)	143 (10.8%)	
	Pancreas	660 (16.9%)	252 (19.1%)	
	Rectum	662 (16.9%)	294 (22.3%)	
	Stomach	373 (9.5%)	132 (10.0%)
	Small intestine	1404 (35.9%)	459 (34.8%)
Prior pancreatic surgery		179 (4.6%)	124 (9.4%)	**<.0001**
Carcinoid syndrome		727 (18.6%)	250 (19.0%)	.75
Prior chemotherapy		593 (15.1%)	232 (17.6%)	**.04**
Prior radiotherapy		238 (6.1%)	80 (6.1%)	.99
Modified Charlson comorbidity index	0	2232 (57.0%)	778 (59.0%)	.21
	1‐2	893 (22.8%)	266 (20.2%)	
	>3	161 (4.1%)	62 (4.7%)	
	Unknown	630 (16.1%)	213 (16.2%)	
Received SSA		544 (13.9%)	240 (18.2%)	**.0002**

**TABLE 3:** Risk factors for developing diabetes mellitus in multivariable analysis

Variable		Hazard ratio	95% confidence interval	*P*‐value
Age	65‐69	Ref.		
	70‐74	0.89	(0.77, 1.04)	.14
	75‐79	0.95	(0.80, 1.12)	.53
	80+	0.85	(0.71, 1.02)	.08
Gender	Male	0.96	(0.85, 1.08)	.46
Race/ethnicity	Non‐Hispanic white	Ref.		
	Non‐Hispanic black	1.58	(1.33,1.89)	**<.0001**
	Hispanic	1.58	(1.25,2.01)	**.0002**
	Asian/Pacific Islander	1.21	(0.95,1.54)	.13
	Other/Unknown	1.15	(0.73,1.80)	.54
Tumor size	<1 cm	Ref.		
	1‐2cm	1.19	(0.97, 1.46)	.09
	>2cm	1.35	(1.09, 1.66)	**.005**
	Unknown	1.15	(0.96, 1.38)	.13
Stage	I	Ref.		
	II	0.93	(0.75, 1.15)	.49
	III	0.84	(0.69, 1.02)	.08
	IV	0.85	(0.70, 1.03)	.09
Histological grade	Well differentiated	Ref.		
	Moderately differentiated	0.97	(0.75, 1.24)	.78
	Poorly differentiated and undifferentiated	1.01	(0.77, 1.33)	.92
	Unknown	1.15	(0.99, 1.33)	.07
Primary site	Rectum	Ref.		
	Colon	0.94	(0.74, 1.20)	.62
	Pancreas	1.49	(1.16, 1.90)	**.002**
	Small intestine	0.92	(0.76, 1.12)	.41
	Stomach	1.03	(0.81, 1.30)	.84
	Appendix	0.79	(0.55, 1.12)	.18
Prior pancreatic surgery		1.35	(1.03, 1.75)	**.03**
Carcinoid syndrome		1.02	(0.88, 1.20)	.76
Prior chemotherapy		1.25	(1.06,1.49)	**.01**
Prior radiotherapy		1.12	(0.88, 1.43)	.36
Modified Charlson comorbidity index	0	Ref.		
	1‐2	1.24	(1.07, 1.44)	**.0054**
	3 or greater	2.40	(1.80, 3.19)	**<.0001**
	Unknown	0.92	(0.77, 1.09)	.33
Received SSA (time‐dependent variable)		1.19	(0.95, 1.49)	.13

Bold values indicates the statistically significant *P*‐values

Tumor factors associated with increased DM risk were primary pancreas tumors (HR 1.49, 95% CI 1.16‐1.90) and tumors larger than 2 cm (HR 1.35, 95% CI 1.09‐1.66). Tumor stage was not associated with development of DM. Likewise, clinical carcinoid syndrome was not significantly associated with the development of DM.

Among previous treatments, prior pancreatic surgery (HR 1.35; 95% CI 1.03‐1.75) and prior chemotherapy (HR 1.25; 95% CI 1.06‐1.49) predisposed patients to developing DM. Higher Charlson comorbidity scores were also associated with increased risk of DM.

**FIGURE 1:**
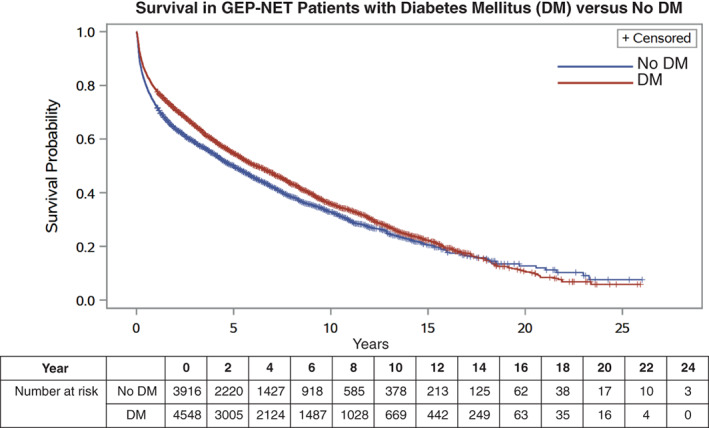
Survival in GEP‐NET patients with diabetes mellitus (DM) vs no DM

Overall survival in all individuals with GEP‐NET was plotted with Kaplan–Meier analysis (Supplementary Figures). In Kaplan–Meier analysis comparing individuals with and without DM, survival in these groups was comparable (Figure 1). However, survival of individuals with pancreatic tumors was significantly less favorable than those with tumors of other primary sites (Supplementary Figures).

## 4 DISCUSSION

Individuals with GEP‐NETs are often treated with SSAs as a first‐line treatment and stay on SSAs for a prolonged period, up to 10 years or more. It is therefore important to understand the long‐term effects of SSAs. Some known potential risks of SSAs include exocrine pancreatic insufficiency (described in 20%‐25% of patients) and biliary stone disease (described in 27% of patients treated with SSA).^19^, ^20^, ^21^ The effects of SSA on pancreatic endocrine function and glycemic control have not yet been elucidated, however, and are especially crucial to explore, given over half of all GEP‐NET patients in our cohort (53%) developed diabetes mellitus in their lifetimes. Although our adjusted analysis demonstrates SSA treatment did not confer increased risk of DM in this cohort, our results show an overall much higher rate of DM in individuals with GEP‐NETs compared to the general US population, in which 21.4% of adults over age 65 are diagnosed with DM.^5^

To our knowledge, the relationship between SSAs and development of DM has not been studied in NET patients. The limited existing literature has shown mixed results on the relationship between SSAs and blood glucose levels. A number of studies in individuals without NETs and animal models have linked SSAs and hyperglycemia. These studies demonstrated that SSA administration results in increased blood glucose and suppressed insulin in rats, and increased blood glucose after sulfonylurea‐induced hypoglycemia in humans.^12^, ^13^, ^22^, ^23^ However clinical trials for octreotide have reported instances of either hyper‐ or hypoglycemia in different individuals.^11^ Still others have reported no significant glucose alterations or only transient changes.^16^, ^24^

This is the first study to describe a markedly higher prevalence of DM in individuals with GEP‐NETs compared to the general population over age 65. One group has previously observed an association between well‐differentiated GEP‐NETs and metabolic syndrome and visceral obesity.^17^, ^18^ Higher fasting glucose and glucose intolerance were described previously in two small samples of patients with NETs; however, the prevalence of DM diagnoses in the GEP‐NET population has not been studied.^17^, ^25^ It has been proposed that DM and neuroendocrine tumors share components in their pathogenesis, though the exact mechanism is not known.^26^ This is supported by studies showing that some medications for diabetes such as metformin have been associated with slower tumor progression in NET patients.^27^, ^28^ Further studies are needed to better explain these relationships.

We found significantly increased risk of diabetes in non‐Hispanic black and Hispanic populations, which is consistent with prior data in both the general population and the elderly.^29^, ^30^, ^31^ A variety of socioeconomic (eg, neighborhood, income) and biologic (eg, BMI, waist circumference) risk factors have been linked to the higher rates of DM in these groups and are likely also at play in our patient population.^32^, ^33^, ^34^ Our finding that pancreatic NETs in particular predicted higher risk of DM has also been described previously, though the direction of causality has been unclear.^35^

Strengths of this study include the examination of an understudied patient population with GEP‐NETs, a large sample size, and low rate of loss to follow‐up. To our knowledge, there have been no prior studies of the relationship between use of SSAs and development of DM in individuals with GEP‐NETs. Limitations of the study include the possibility of selection bias. Only patients over 65 and therefore eligible for Medicare were included, and this older sample may not represent all individuals with GEP‐NETs. However, this study's older population is an important population to understand, in light of prolonged survival of patients with GEP‐NETs, as well as the aging of the US population as a whole. Additionally, the rate of SSA treatment was 15% of our sample, lower than expected given the largely well‐differentiated and late‐stage disease in this sample, which would likely be good candidates for treatment. This raises the possibility that SSA treatment data may be incompletely captured or underestimated in the SEER‐Medicare database, or may represent differences in treatment practices over time. Low SSA treatment rates were similarly seen in a prior SEER‐Medicare analysis of all nonpancreatic GI NET patients, in which just 6% of patients received SSA.^4^

Overall, our study shows a substantially higher rate of DM in individuals with GEP‐NETs than in the general population. As patients with GEP‐NETs have increasingly favorable cancer‐related survival, it is important for clinicians to understand these patients' increased risk of DM, given the significant morbidity and mortality of DM over time. Though some prior literature suggests SSAs are linked to hyperglycemia and glucose dysregulation, SSAs were not associated with increased risk of DM in our sample. Further studies are needed to better understand the relationship between glucose regulation, SSA treatment, and the increased DM risk in individuals with GEP‐NETs.

## AUTHOR CONTRIBUTIONS

**Katherine Ni:** Conceptualization; data curation; formal analysis; investigation; methodology; software; visualization; writing‐original draft; writing‐review and editing. **Jeong Yun Yang:** Data curation; formal analysis; methodology; software; writing‐review and editing. **Kiwoon Baeg:** Data curation; formal analysis; methodology; software; writing‐review and editing. **Amanda Leiter:** Methodology; validation; writing‐review and editing. **Grace Mhango:** Data curation; methodology; software; writing‐review and editing. **Emily Gallagher:** Conceptualization; validation; writing‐review and editing. **Juan Wisnivesky:** Conceptualization; formal analysis; methodology; software; supervision; validation; writing‐review and editing. **Michelle Kim:** Conceptualization; data curation; formal analysis; funding acquisition; investigation; methodology; project administration; resources; supervision; validation; writing‐review and editing.

## CONFLICT OF INTEREST

Author J.W. received consulting honorarium from Sanofi, Banook, and GSK, and a research grant from Sanofi. Author E.G. has an advisory role for Novartis and consulting role for Seattle Genetics. The other authors declare no potential conflicts of interest.

## ETHICS STATEMENT

This study was approved by the Icahn School of Medicine Institutional Review Board: Study IRB‐20‐03072. This study has been done in accordance to the Publishing Ethics Guidelines outlined by the Committee on Publication Ethics (COPE).

## Supporting information


Supplementary Figures

## Data Availability

The data that support the findings of this study are available from the CMS Information Management Services and the Surveillance, Epidemiology, and End Results (SEER) Program tumor registries. Restrictions apply to the availability of these data, which were used under license for this study. Data are available from https://healthcaredelivery.cancer.gov/seermedicare/ with the permission.

## References

[1] Yao JC , Hassan M , Phan A , et al. One hundred years after "carcinoid": epidemiology of and prognostic factors for neuroendocrine tumors in 35,825 cases in the United States. J Clin Oncol. 2008;26(18):3063‐3072. 18565894 10.1200/JCO.2007.15.4377

[2] Dasari A , Shen C , Halperin D , et al. Trends in the incidence, prevalence, and survival outcomes in patients with neuroendocrine tumors in the United States. JAMA Oncol. 2017;3:1335‐1342. 28448665 10.1001/jamaoncol.2017.0589 PMC5824320

[3] Kim MK , Warner RR , Roayaie S , et al. Revised staging classification improves outcome prediction for small intestinal neuroendocrine tumors. J Clin Oncol. 2013;31(30):3776‐3781. 24043726 10.1200/JCO.2013.51.1477 PMC3906571

[4] Halperin DM , Shen C , Dasari A , et al. Frequency of carcinoid syndrome at neuroendocrine tumour diagnosis: a population‐based study. Lancet Oncol. 2017;18(4):525‐534. 28238592 10.1016/S1470-2045(17)30110-9 PMC6066284

[5] National Diabetes Statistics Report . Centers for Disease Control and Prevention. Translation DoD. 2020;2020:2.

[6] American Diabetes A . 11. Microvascular complications and foot care: standards of medical care in diabetes‐2019. Diabetes Care. 2019;42(Suppl 1):S124‐S138. 30559237 10.2337/dc19-S011

[7] American Diabetes A . 10. Cardiovascular disease and risk management: standards of medical Care in Diabetes‐2019. Diabetes Care. 2019;42(Suppl 1):S103‐S123. 30559236 10.2337/dc19-S010

[8] Gregg EW , Gu Q , Cheng YJ , Narayan KM , Cowie CC . Mortality trends in men and women with diabetes, 1971 to 2000. Ann Intern Med. 2007;147(3):149‐155. 17576993 10.7326/0003-4819-147-3-200708070-00167

[9] Mulnier HE , Seaman HE , Raleigh VS , Soedamah‐Muthu SS , Colhoun HM , Lawrenson RA . Mortality in people with type 2 diabetes in the UK. Diabet Med. 2006;23(5):516‐521. 16681560 10.1111/j.1464-5491.2006.01838.x

[10] American Diabetes A . 4. Comprehensive medical evaluation and assessment of comorbidities: standards of medical care in diabetes‐2019. Diabetes Care. 2019;42(Suppl 1):S34‐S45. 30559230 10.2337/dc19-S004

[11] Sandostatin Full Prescribing Information. East Hanover, NJ: Novartis Pharmaceuticals Corporation; 2019.

[12] Fasano CJ , O'Malley G , Dominici P , Aguilera E , Latta DR . Comparison of octreotide and standard therapy versus standard therapy alone for the treatment of sulfonylurea‐induced hypoglycemia. Ann Emerg Med. 2008;51(4):400‐406. 17764782 10.1016/j.annemergmed.2007.06.493

[13] Glatstein M , Scolnik D , Bentur Y . Octreotide for the treatment of sulfonylurea poisoning. Clin Toxicol (Philadelphia, PA). 2012;50(9):795‐804. 10.3109/15563650.2012.734626 23046209

[14] Plöckinger U , Holst JJ , Messerschmidt D , Hopfenmüller W , Quabbe HJ . Octreotide suppresses the incretin glucagon‐like peptide (7‐36) amide in patients with acromegaly or clinically nonfunctioning pituitary tumors and in healthy subjects. Eur J Endocrinol. 1999;140(6):538‐544. 10377503 10.1530/eje.0.1400538

[15] Davies RR , Turner SJ , Alberti KG , Johnston DG . Somatostatin analogues in diabetes mellitus. Diabet Med. 1989;6(2):103‐111. 2564819 10.1111/j.1464-5491.1989.tb02096.x

[16] Orskov L , Møller N , Bak JF , Pørksen N , Schmitz O . Effects of the somatostatin analog, octreotide, on glucose metabolism and insulin sensitivity in insulin‐dependent diabetes mellitus. Metabol Clin Exp. 1996;45(2):211‐217. 10.1016/s0026-0495(96)90056-6 8596492

[17] Santos AP , Castro C , Antunes L , Henrique R , Cardoso MH , Monteiro MP . Disseminated well‐differentiated gastro‐Entero‐pancreatic tumors are associated with metabolic syndrome. J Clin Med. 2019;8(9):1479. 10.3390/jcm8091479 PMC6780069 31533348

[18] Santos AP , Santos AC , Castro C , et al. Visceral obesity and metabolic syndrome are associated with well‐differentiated gastroenteropancreatic neuroendocrine tumors. Cancers. 2018;10(9):293. 10.3390/cancers10090293 PMC6162651 30150555

[19] Rinzivillo M , De Felice I , Magi L , Annibale B , Panzuto F . Occurrence of exocrine pancreatic insufficiency in patients with advanced neuroendocrine tumors treated with somatostatin analogs. Pancreatology. 2020;20(5):875‐879. 32684368 10.1016/j.pan.2020.06.007

[20] Lamarca A , McCallum L , Nuttall C , et al. Somatostatin analogue‐induced pancreatic exocrine insufficiency in patients with neuroendocrine tumors: results of a prospective observational study. Exp Rev Gastroenterol Hepatol. 2018;12(7):723‐731. 10.1080/17474124.2018.1489232 29923433

[21] Brighi N , Panzuto F , Modica R , et al. Biliary stone disease in patients with neuroendocrine tumors treated with Somatostatin analogs: a multicenter study. Oncologist. 2020;25(3):259‐265. 32162819 10.1634/theoncologist.2019-0403 PMC7066710

[22] Griffiths J , Mills MT , Ong AC . Long‐acting somatostatin analogue treatments in autosomal dominant polycystic kidney disease and polycystic liver disease: a systematic review and meta‐analysis. BMJ Open. 2020;10(1):e032620. 10.1136/bmjopen-2019-032620 PMC6955551 31924636

[23] Daumerie C , Henquin JC . Somatostatin and the intestinal transport of glucose and other nutrients in the anaesthetised rat. Gut. 1982;23(2):140‐145. 6121743 10.1136/gut.23.2.140 PMC1419551

[24] Rinke A , Wittenberg M , Schade‐Brittinger C , et al. Placebo‐controlled, double‐blind, prospective, randomized study on the effect of Octreotide LAR in the control of tumor growth in patients with metastatic neuroendocrine Midgut tumors (PROMID): results of long‐term survival. Neuroendocrinology. 2017;104(1):26‐32. 26731483 10.1159/000443612

[25] Feldman JM , Plonk JW , Bivens CH , Lebovitz HE . Glucose intolerance in the carcinoid syndrome. Diabetes. 1975;24(7):664‐671. 125668 10.2337/diab.24.7.664

[26] Herold Z , Doleschall M , Kovesdi A , Patocs A , Somogyi A . Chromogranin A and its role in the pathogenesis of diabetes mellitus. Endokrynol Pol. 2018;69(5):598‐610. 30074235 10.5603/EP.a2018.0052

[27] Pusceddu S , Vernieri C , Di Maio M , et al. Metformin use is associated with longer progression‐free survival of patients with diabetes and pancreatic neuroendocrine tumors receiving Everolimus and/or Somatostatin analogues. Gastroenterol. 2018;155(2):479‐89.e7. 10.1053/j.gastro.2018.04.010 29655834

[28] Herrera‐Martínez AD , Pedraza‐Arevalo S , López FL , et al. Type 2 diabetes in neuroendocrine tumors: are Biguanides and statins part of the solution? J Clin Endocrinol Metab. 2019;104(1):57‐73. 30265346 10.1210/jc.2018-01455

[29] Walker RJ , Strom Williams J , Egede LE . Influence of race, ethnicity and social determinants of health on diabetes outcomes. Am J Med Sci. 2016;351(4):366‐373. 27079342 10.1016/j.amjms.2016.01.008 PMC4834895

[30] Geiss LS , Wang J , Cheng YJ , et al. Prevalence and incidence trends for diagnosed diabetes among adults aged 20 to 79 years, United States, 1980‐2012. JAMA. 2014;312(12):1218‐1226. 25247518 10.1001/jama.2014.11494

[31] Kulick ER , Moon YP , Cheung K , Willey JZ , Sacco RL , Elkind MS . Racial‐ethnic disparities in the association between risk factors and diabetes: the northern Manhattan study. Prev Med. 2016;83:31‐36. 26658025 10.1016/j.ypmed.2015.11.023 PMC4724287

[32] McBean AM , Li S , Gilbertson DT , Collins AJ . Differences in diabetes prevalence, incidence, and mortality among the elderly of four racial/ethnic groups: whites, blacks, hispanics, and asians. Diabetes Care. 2004;27(10):2317‐2324. 15451894 10.2337/diacare.27.10.2317

[33] Piccolo RS , Subramanian SV , Pearce N , Florez JC , McKinlay JB . Relative contributions of socioeconomic, local environmental, psychosocial, lifestyle/behavioral, biophysiological, and ancestral factors to racial/ethnic disparities in type 2 diabetes. Diabetes Care. 2016;39(7):1208‐1217. 27330127 10.2337/dc15-2255 PMC4915558

[34] Chatterjee R , Brancati FL , Shafi T , et al. Non‐traditional risk factors are important contributors to the racial disparity in diabetes risk: the atherosclerosis risk in communities study. J Gen Intern Med. 2014;29(2):290‐297. 23943422 10.1007/s11606-013-2569-z PMC3912297

[35] Gallo M , Ruggeri RM , Muscogiuri G , Pizza G , Faggiano A , Colao A . Diabetes and pancreatic neuroendocrine tumours: which interplays, if any? Cancer Treat Rev. 2018;67:1‐9. 29746922 10.1016/j.ctrv.2018.04.013

