# Supplementary figures and images for: Association between somatostatin analogues and diabetes mellitus in gastroenteropancreatic neuroendocrine tumor patients: A Surveillance, Epidemiology, and End Results‐Medicare analysis of 5235 patients
Source: Cancer Rep (Hoboken). 2021 Apr 9;4(5):e1387. doi: 10.1002/cnr2.1387 (PMC8551991; doi:10.1002/cnr2.1387)

Supplemental Figure A.


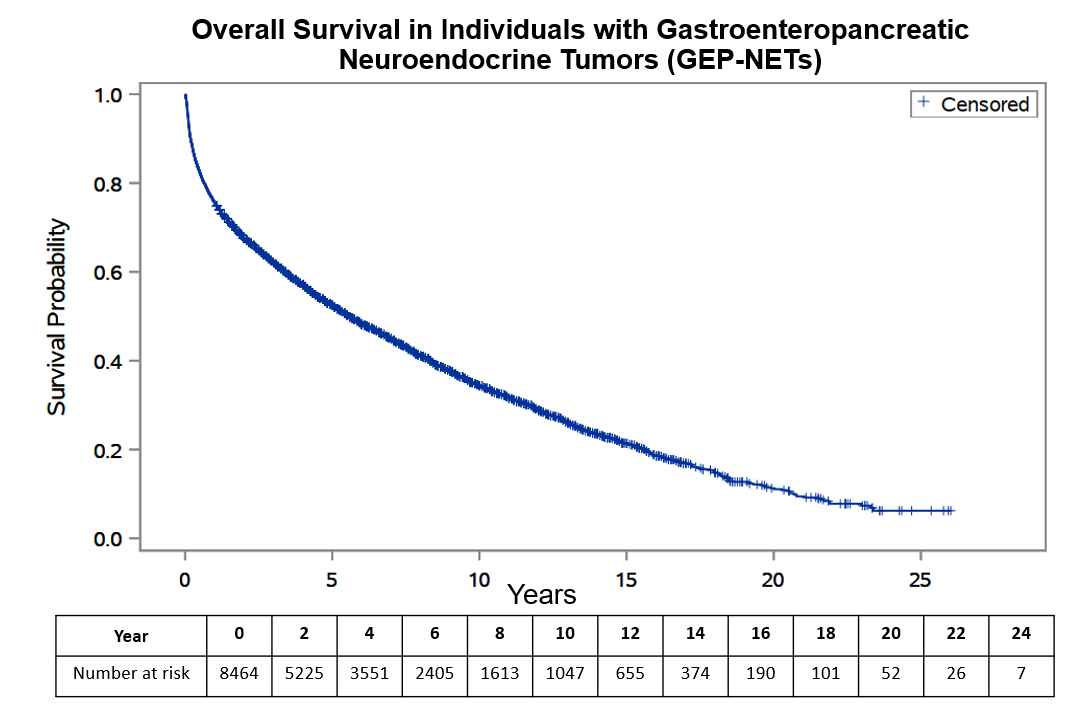


Supplemental Figure B.


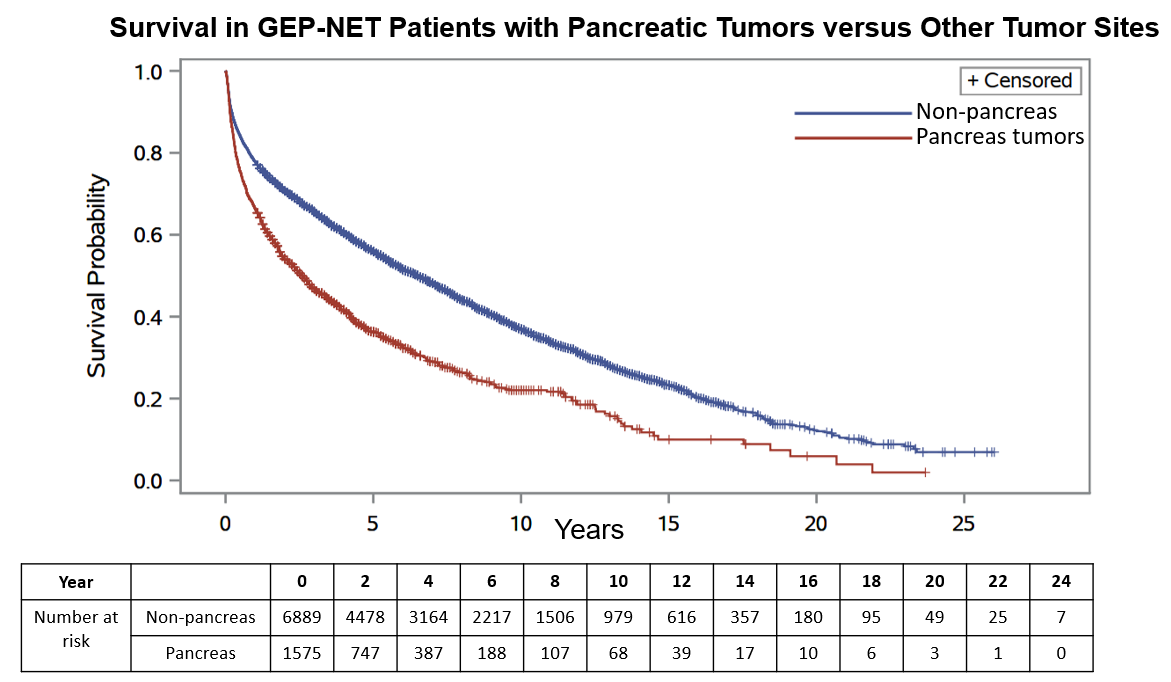

Supplement: Supplementary file 1 — Supplementary Figures [file CNR2-4-e1387-s001.docx]
